# Season of Birth Predicts Emotional and Behavioral Regulation in 18-Month-Old Infants: Hamamatsu Birth Cohort for Mothers and Children (HBC Study)

**DOI:** 10.3389/fpubh.2016.00152

**Published:** 2016-07-25

**Authors:** Ryosuke Asano, Kenji J. Tsuchiya, Taeko Harada, Yumeno Kugizaki, Ryuji Nakahara, Chikako Nakayasu, Akemi Okumura, Yukiko Suzuki, Shu Takagai, Norio Mori, Nori Takei, for Hamamatsu Birth Cohort (HBC) Study Team

**Affiliations:** ^1^Research Center for Child Mental Development, Hamamatsu University School of Medicine, Hamamatsu, Japan; ^2^Department of Child Development, United Graduate School of Child Development, Osaka University, Kanazawa University, Hamamatsu University School of Medicine, Chiba University and University of Fukui, Hamamatsu, Japan; ^3^Department of Psychiatry, Hamamatsu University School of Medicine, Hamamatsu, Japan; ^4^Division of Psychological Medicine, Institute of Psychiatry, King’s College London, London, UK

**Keywords:** effortful control, aggression, infancy, season of birth, birth cohort, Japan, structural equation modeling

## Abstract

**Background:**

Previous research has demonstrated that the season of birth may predict development of emotional and behavioral regulation during childhood or adolescence. This study examined whether the season of birth predicts effortful control (i.e., the ability to voluntarily choose course of actions during conflict and to plan for the future) and aggression (i.e., the use of physical force and expression of anger toward others) in 18-month-old infants.

**Methods:**

Participants included 885 infants who were enrolled in the Hamamatsu Birth Cohort for Mothers and Children in Hamamatsu, Japan. Seasons of birth were categorized into winter (December, January, and February), spring (March, April, and May), summer (June, July, and August), and autumn (September, October, and November). At 18 months of age, effortful control was assessed using the Early Childhood Behavior Questionnaire, and aggression was measured using the Cardiff Infant Contentiousness Scale. Structural equation modeling analysis with measurement and structural equations was conducted to test our prediction.

**Results:**

Effortful control was higher in infants born in spring [*B* = 0.095, 95% CI (0.014 to 0.175), *p* = 0.021, β = 0.146] and summer [*B* = 0.078, 95% CI (0.001 to 0.156), *p* = 0.049, β = 0.118] than in those born in winter. In addition, aggression was lower in those born in spring [*B* = −0.286, 95% CI (−0.551 to −0.021), *p* = 0.035, β = −0.135] than those born in winter, even after controlling for seven covariates.

**Conclusion:**

The findings suggest that season of birth may determine development of emotional and behavioral regulation skills during early infancy. Future research should pay more attention to the underlying mechanisms of the effects of birth season on development of emotional and behavioral regulation during infancy.

## Introduction

Previous studies have suggested that higher effortful control and lower aggression represent greater emotional and behavioral regulation skills. Effortful control refers to the ability to voluntarily choose a course of action under conditions of conflict and to plan for the future and detect errors. This includes maintaining attention to a particular activity, transferring attention to other activities, and controlling behavior under instruction (1). In contrast, aggression refers to the use of physical force and expression of anger directed at family members and peers (2). Findings revealed that effortful control and aggression emerge approximately at 1–3 years of age (3–5). In addition, it is possible that lower effortful control and higher aggression during infancy lead to antisocial behavior in childhood and later in life (3, 6, 7). Therefore, to support healthy development of children, it is important to understand the factors that influence effortful control and aggression, representing levels of emotional and behavioral regulation during early infancy.

Increasingly, the literature has demonstrated that season of birth may predict development of emotional and behavioral regulation during childhood or adolescence. Chotai et al. (8, 9) investigated the association between season of birth and temperament in a sample population in Sweden. The study found that adults, particularly women, who were born in summer had higher levels of temperament trait for novelty seeking and tended to show more interest in excitement, impulsiveness, extravagance, and disorderliness (10) than those born during the rest of the year. On the contrary, two other studies found that adolescent and young adult women who were born in winter displayed higher levels of novelty seeking than those born during the other three seasons (9, 11). It was also reported that adolescents in Japan who were born in winter had lower levels of agreeableness, which is one of the “Big Five” personality traits and is associated with greater anger and hostility (12), than those born during the rest of the year (13). Furthermore, university students in Hungary who were born in winter had lower levels of irritable temperament than those born during other seasons (14).

Although informative, these studies have mainly focused on children and adolescents and need to be extended to early infants, in order to understand development of emotional and behavioral regulation skills. If the particular season of birth decreases effortful control and increases aggression during infancy, these emotional and behavioral aspects of personality may increase antisocial behavior both in childhood and later in life (3, 6, 7). Therefore, we illuminate the predictive power of birth season on effortful control and aggression during infancy for early prediction and prevention of antisocial behavior later in life.

This study was conducted to examine whether season of birth affects emotional and behavioral regulation skills – higher effortful control and lower aggression – during early infancy. To this end, we recruited infants at 18 months of age using a representative birth cohort in Japan.

## Materials and Methods

This study was conducted as part of the “Hamamatsu Birth Cohort for Mothers and Children (HBC Study).” The HBC Study has been described in detail elsewhere (15, 16) and is an ongoing cohort study in Hamamatsu, Japan. Pregnant women and their partners who were given a complete description of the study and agreed to participate in the study were enrolled and provided written informed consent. Once during the second trimester of pregnancy and 12 times in 8 years after delivery, we performed detailed face-to-face interviews and direct assessments in our two research sites: the Hamamatsu University Hospital and Kato Maternity Clinic. We had previously established that the participants of the HBC Study were representative of Japanese mothers in terms of age, socioeconomic status, history of major depressive disorders, and parity. Furthermore, the children were representative of Japanese children for birth weight and gestational age (15, 16).

### Participants

We recruited all the pregnant women who were expected to give birth at the research sites and had a delivery between December 2007 and March 2011. Participants included 1,138 mothers who were first enrolled in the HBC Study and their infants aged 18 months. To keep all participants as independent observations, we excluded 19 mothers who delivered twins from the following analyses. Consequently, among 1,119 participants, 234 infants were not assessed for effortful control and aggression because they were not in contact with our research center or had canceled assessment at the age of 18 months due to poor health, a residential move, or death.

Our final sample comprised 885 infants. The following values were derived for the group of infants included (*n* = 885) and excluded (*n* = 253) in the analysis: mean age of mother (31.1 vs. 29.9 years), mean age of father (33.0 vs. 31.7 years), average household income (6.17 vs. 5.60 million JPY), maternal history of depression and/or anxiety disorder (yes 12.1 vs. 11.4%), gender of child (male 49.9 vs. 52.2%), older siblings (yes 54.6 vs. 51.4%), influenza by the age of 18 months (yes 8.5 vs. 0.0%), and food allergy by the age of 18 months (yes 8.6 vs. 0.0%).

### Measures

We assessed emotional and behavioral regulation skills by two scales at 18 months of age. First, effortful control was assessed using the Early Childhood Behavior Questionnaire [ECBQ; (17)], which is a measure assessing temperament in children aged approximately 1–3 years. Effortful control consists of five dimensions: attention focusing (12 items; e.g., “When playing alone, how often did your child play with a set of objects for 5 min or longer at a time?”), attention shift (12 items; e.g., “During everyday activities, how often did your child seem able to easily shift attention from one activity to another?”), inhibitory control (12 items; e.g., “When told “no,” how often did your child stop an activity quickly?”), low-intensity pleasure (11 items; e.g., “During daily or evening quiet time with you and your child, how often did your child enjoy just being quietly sung to?”), and cuddliness (12 items; e.g., “When being gently rocked or hugged, how often did your child seem eager to get away?”). McDonald’s ω coefficients were 0.92, 0.77, 0.87, 0.78, and 0.92, respectively, indicating higher internal consistency reliability of each dimension. Interviewers rated the degree to which each statement applied to the participating infants using a five-point scale from 1 = *never* to 5 = *always* via face-to-face interviews with their caregivers. Ratings were averaged to yield each subscale score. Higher scores represent greater levels of effortful control during early infancy, thus, indicating better emotional and behavioral regulation skills.

Second, aggression was assessed using the Cardiff Infant Contentiousness Scale (CICS) (2). This is a measure assessing the use of physical force and expression of anger directed at family members and peers in children aged approximately 6 months–3 years. The CICS contains the following four items: “bites,” “hits out,” “has angry moods,” and “has temper tantrums.” Interviewers rated the degree to which each statement applied to the participating infants using a three-point scale from 0 = *absent* to 2 = *definitely present* via face-to-face interviews with their caregivers. Higher scores represent greater levels of aggression during early infancy, indicating less emotional and behavioral regulation skills.

As with previous research based on the HBC Study (18), the 12 months of the year of birth were categorized into four seasons: winter (December, January, and February), spring (March, April, and May), summer (June, July, and August), and autumn (September, October, and November).

For covariates, we opted for the following seven variables that have been reported to be associated with effortful control and aggression in the literature (3, 19) and were available in the HBC Study: maternal and paternal age, annual household income, maternal history of depression and/or anxiety disorders, infantile gender, and older brothers and sisters. The maternal history of depression and/or anxiety disorders was evaluated by trained interviewers using the Structured Clinical Interview for DSM-IV Axis I Disorders (20), as in previous studies (21, 22). All covariates were assessed during the second trimester of pregnancy, except for infantile gender.

### Ethical Statement

The study protocol was approved by Hamamatsu University School of Medicine and the University Hospital Ethics Committee (No. 20-82, 21-114, 22-29, 24-67, 24-237, 25-143, 25-283, E14-062).

### Statistical Analysis

**Figure 1:**
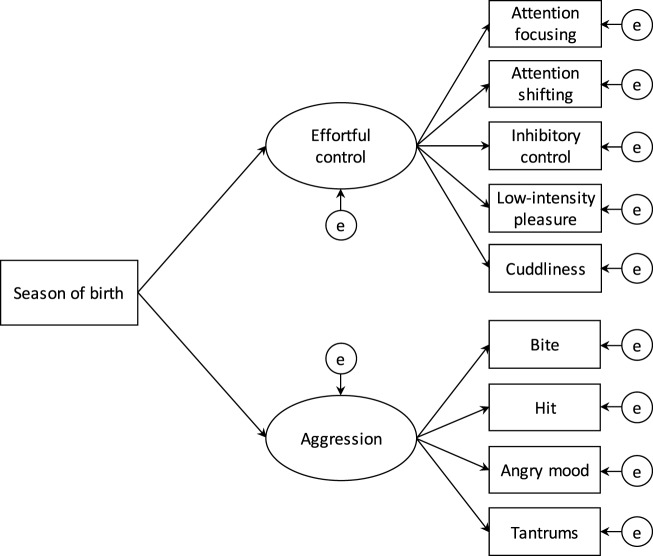
**Structural equation modeling for the effects of birth season on effortful control and aggression in 18-month-old infants**.

First, we calculated the number and proportion of season (and month) of birth and examined descriptive statistics for subscales of effortful control and aggression by season (and month) of birth among early infants. Second, we conducted a series of structural equation modeling analyses with measurement and structural equations to test the effects of season of birth on effortful control and aggression in 18-month-old infants (Figure 1). Crude analyses did not incorporate an adjustment for any covariates, and analyses allowing for covariates included maternal and paternal age, annual household income, maternal history of depression and/or anxiety disorders, infantile gender, and older brothers and sisters. In addition to season of birth, we performed supplementary analysis to examine the effects of month of birth on effortful control and aggression in 18-month-old infants. The strength of effects of season (and month) of birth on effortful control and aggression were evaluated using partial regression coefficients *B* (i.e., the estimated effect of the predictor by one unit on the outcomes when simultaneously considering other predictors) and standardized partial regression coefficients β (i.e., the estimated effect of the predictor by one standard deviation on the outcomes when simultaneously considering other predictors, ranging from −1 to 1).

Stata, version 14.0 (23) was used for preliminary analyses, and Mplus 7.4 (24) was used for the structural equation modeling analyses. We considered *p*-values of <0.05 to be statistically significant.

## Results

### Characteristics of Participants

Table 1 shows the number and proportion of predictor variables and covariates in each subcategory of season (and month) of birth, and Table 2 indicates descriptive statistics for effortful control and aggression by season (and month) of birth. There were no significant differences between observed frequencies and expected frequencies for season of birth [χ^2^ (3) = 0.431, *p* = 0.934], indicating that season of birth among the participating infants were equally distributed.

**Table 1:** **Number and proportion of predictors and covariates (*N* = 885)**.

Season of birth	Winter (December–February)	222 (25.1%)
	Spring (March–May)	229 (25.9%)
	Summer (June–August)	217 (24.5%)
	Autumn (September–November)	217 (24.5%)
Month of birth	January	82 (9.3%)
	February	79 (8.9%)
	March	84 (9.5%)
	April	79 (8.9%)
	May	66 (7.5%)
	June	81 (9.2%)
	July	81 (9.2%)
	August	55 (6.2%)
	September	86 (9.7%)
	October	68 (7.7%)
	November	63 (7.1%)
	December	61 (6.9%)
Maternal age at birth	<25 years	80 (9.0%)
	25–29 years	259 (29.3%)
	30–34 years	303 (34.2%)
	≥35 years	243 (27.5%)
Paternal age at birth	<25 years	49 (5.5%)
	25–29 years	196 (22.2%)
	30–34 years	317 (35.8%)
	≥35 years	323 (36.5%)
Annual household income	≥8 million yen	182 (20.6%)
	3–7.9 million yen	660 (74.6%)
	<3 million yen	43 (4.9%)
Maternal history of depression and/or anxiety disorder	No	779 (88.0%)
	Yes	106 (12.0%)
Infantile gender	Female	443 (50.1%)
	Male	442 (49.9%)
Older brothers	Yes	240 (27.1%)
	No	645 (72.9%)
Older sisters	Yes	217 (24.5%)
	No	668 (75.5%)

**Table 2:** **Means and SDs for subscales of effortful control and aggression by season and month of birth (*N* = 885)**.

	Effortful control					Aggression			
	Attention focusing	Attention shifting	Inhibitory control	Low-intensity pleasure	Cuddliness	Bite	Hit	Angry mood	Tantrums
**Season of birth**									
Winter (December–February)	2.72 (0.69)	3.38 (0.54)	2.37 (0.60)	3.42 (0.52)	3.04 (0.69)	0.38 (0.67)	0.75 (0.81)	0.16 (0.44)	0.69 (0.80)
Spring (March–May)	2.85 (0.72)	3.44 (0.47)	2.50 (0.59)	3.47 (0.49)	3.17 (0.71)	0.33 (0.64)	0.62 (0.81)	0.13 (0.39)	0.55 (0.77)
Summer (June–August)	2.92 (0.73)	3.39 (0.48)	2.44 (0.61)	3.45 (0.48)	3.20 (0.69)	0.21 (0.50)	0.81 (0.85)	0.21 (0.52)	0.64 (0.80)
Autumn (September–November)	2.93 (0.71)	3.35 (0.51)	2.43 (0.57)	3.44 (0.42)	3.05 (0.65)	0.31 (0.64)	0.68 (0.81)	0.17 (0.44)	0.61 (0.76)
**Month of birth**									
January	2.70 (0.67)	3.32 (0.58)	2.44 (0.62)	3.34 (0.56)	2.95 (0.68)	0.32 (0.61)	0.85 (0.79)	0.10 (0.34)	0.85 (0.83)
February	2.79 (0.70)	3.46 (0.51)	2.32 (0.55)	3.49 (0.52)	3.16 (0.75)	0.46 (0.75)	0.77 (0.85)	0.16 (0.46)	0.63 (0.79)
March	2.84 (0.66)	3.35 (0.45)	2.40 (0.61)	3.44 (0.49)	3.12 (0.67)	0.42 (0.73)	0.82 (0.85)	0.14 (0.41)	0.70 (0.83)
April	2.80 (0.72)	3.51 (0.50)	2.53 (0.61)	3.52 (0.49)	3.15 (0.77)	0.33 (0.63)	0.51 (0.77)	0.13 (0.43)	0.42 (0.71)
May	2.94 (0.79)	3.48 (0.42)	2.60 (0.53)	3.46 (0.48)	3.23 (0.67)	0.23 (0.52)	0.50 (0.75)	0.11 (0.31)	0.50 (0.73)
June	2.81 (0.72)	3.35 (0.53)	2.51 (0.61)	3.39 (0.48)	3.16 (0.71)	0.21 (0.52)	0.73 (0.82)	0.16 (0.43)	0.58 (0.77)
July	2.94 (0.75)	3.42 (0.45)	2.40 (0.61)	3.40 (0.48)	3.21 (0.66)	0.26 (0.54)	0.90 (0.87)	0.23 (0.55)	0.74 (0.82)
August	3.04 (0.72)	3.43 (0.44)	2.41 (0.59)	3.62 (0.45)	3.26 (0.72)	0.13 (0.39)	0.78 (0.88)	0.25 (0.58)	0.58 (0.81)
September	2.90 (0.70)	3.33 (0.50)	2.53 (0.53)	3.45 (0.41)	3.13 (0.67)	0.19 (0.52)	0.52 (0.79)	0.16 (0.43)	0.64 (0.77)
October	2.97 (0.78)	3.31 (0.55)	2.37 (0.60)	3.41 (0.38)	3.07 (0.54)	0.40 (0.74)	0.79 (0.84)	0.13 (0.34)	0.53 (0.76)
November	2.92 (0.65)	3.41 (0.47)	2.35 (0.58)	3.45 (0.49)	2.92 (0.70)	0.40 (0.66)	0.78 (0.79)	0.22 (0.55)	0.67 (0.76)
December	2.66 (0.73)	3.36 (0.53)	2.35 (0.65)	3.45 (0.47)	2.99 (0.60)	0.36 (0.66)	0.59 (0.76)	0.23 (0.53)	0.56 (0.76)

*Values in parentheses are SDs. Subscales of effortful control range from 1 to 5; items of aggression range from 0 to 2*.

### Does Season of Birth Predict Effortful Control and Aggression?

The adjustment model revealed that, even after controlling for covariates (Table 3), being born in spring was positively associated with effortful control [*B* = 0.095, 95% CI (0.014 to 0.175), *p* = 0.021, β = 0.146] and negatively associated with aggression [*B* = −0.286, 95% CI (−0.551 to −0.021), *p* = 0.035, β = −0.135]. This suggests that early infants who were born in spring had higher effortful control and lower aggression than those born in winter. In addition, those born in summer were positively associated with effortful control [*B* = 0.078, 95% CI (0.001 to 0.156), *p* = 0.049, β = 0.118], indicating that early infants who were born in summer had higher effortful control than those born in winter.

**Table 3:** **Predicting latent factors of effortful control and aggression by season of birth (*N* = 885)**.

	Crude^a^				Adjustment^b^			
	*B*	95% CI	*p*	β	*B*	95% CI	*p*	β
**Season of birth**	**Effortful control (*R*^2^ = 0.017)**				**Effortful control (*R*^2^ = 0.070)**			
Winter (December–February)	Ref				Ref			
Spring (March–May)	0.090	0.010 to 0.170	0.027	0.145	0.095	0.014 to 0.175	0.021	0.146
Summer (June–August)	0.072	−0.004 to 0.148	0.065	0.113	0.078	0.001 to 0.156	0.049	0.118
Autumn (September–November)	0.032	−0.041 to 0.105	0.393	0.050	0.029	−0.046 to 0.105	0.450	0.044
**Season of birth**	**Aggression (*R*^2^ = 0.011)**				**Aggression (*R*^2^ = 0.086)**			
Winter (December–February)	Ref				Ref			
Spring (March–May)	−0.267	−0.530 to −0.003	0.047	−0.126	−0.286	−0.551 to −0.021	0.035	−0.135
Summer (June–August)	−0.082	−0.327 to 0.162	0.508	−0.038	–0.115	−0.358 to 0.129	0.356	−0.053
Autumn (September–November)	−0.135	−0.364 to 0.095	0.250	−0.062	−0.166	−0.398 to 0.065	0.159	−0.077

*^a^No adjustment made for potential confounders*.

*^b^Adjusted for maternal and paternal age, annual household income, maternal history of depression and/or anxiety disorders, infantile gender, older brothers, and sisters*.

### Does Month of Birth Predict Effortful Control and Aggression?

As seen in Table 4, the adjustment model showed that early infants who were born in February [*B* = 0.122, 95% CI (0.003 to 0.242), *p* = 0.045, β = 0.126], April [*B* = 0.173, 95% CI (0.047 to 0.298), *p* = 0.007, β = 0.178], May [*B* = 0.194, 95% CI (0.056 to 0.332), *p* = 0.006, β = 0.185], August [*B* = 0.214, 95% CI (0.086 to 0.342), *p* = 0.001, β = 0.187], and September [*B* = 0.123, 95% CI (0.007–0.239), *p* = 0.037, β = 0.133] had higher effortful control than those born in January. In addition, early infants who were born in April [*B* = −0.539, 95% CI (−0.953 to −0.126), *p* = 0.011, β = −0.184], May [*B* = −0.625, 95% CI (−1.053 to −0.197), *p* = 0.004, β = −0.197], and September [*B* = −0.545, 95% CI (−0.884 to −0.206), *p* = 0.002, β = −0.193] had lower aggression than those born in January.

**Table 4:** **Predicting latent factors of effortful control and aggression by month of birth (*N* = 885)**.

	Crude^a^				Adjustment^b^			
	*B*	95% CI	*p*	β	*B*	95% CI	*p*	β
**Month of birth**	**Effortful control (*R*^2^ = 0.042)**				**Effortful control (*R*^2^ = 0.097)**			
January	Ref				Ref			
February	0.105	−0.011 to 0.222	0.076	0.114	0.122	0.003 to 0.242	0.045	0.126
March	0.075	−0.037 to 0.187	0.190	0.084	0.086	−0.031 to 0.203	0.150	0.091
April	0.163	0.039 to 0.286	0.010	0.176	0.173	0.047 to 0.298	0.007	0.178
May	0.171	0.035 to 0.307	0.014	0.170	0.194	0.056 to 0.332	0.006	0.185
June	0.080	−0.037 to 0.196	0.180	0.087	0.092	−0.027 to 0.211	0.132	0.096
July	0.094	−0.022 to 0.210	0.112	0.103	0.115	−0.003 to 0.233	0.057	0.120
August	0.201	0.078 to 0.325	0.001	0.185	0.214	0.086 to 0.342	0.001	0.187
September	0.105	−0.007 to 0.217	0.066	0.118	0.123	0.007 to 0.239	0.037	0.133
October	0.054	−0.061 to 0.169	0.355	0.055	0.051	−0.068 to 0.170	0.399	0.050
November	0.064	−0.062 to 0.190	0.320	0.062	0.063	−0.070 to 0.196	0.353	0.059
December	0.036	−0.090 to 0.161	0.575	0.035	0.045	−0.082 to 0.171	0.489	0.041
**Month of birth**	**Aggression (*R*^2^ = 0.057)**				**Aggression (*R*^2^ = 0.129)**			
January	Ref				Ref			
February	−0.110	−0.464 to 0.244	0.543	−0.037	−0.144	−0.473 to 0.186	0.393	−0.049
March	−0.052	−0.376 to 0.272	0.753	−0.019	−0.100	−0.402 to 0.202	0.517	−0.035
April	−0.568	−1.007 to −0.130	0.011	−0.188	−0.539	−0.953 to −0.126	0.011	−0.184
May	−0.598	−1.046 to −0.150	0.009	−0.177	−0.625	−1.053 to −0.197	0.004	−0.197
June	−0.307	−0.674 to 0.060	0.101	−0.105	−0.303	−0.644 to 0.037	0.081	−0.105
July	−0.013	−0.331 to 0.306	0.938	−0.001	−0.098	−0.393 to 0.198	0.518	−0.034
August	−0.306	−0.718 to 0.106	0.146	−0.088	−0.323	−0.711 to 0.065	0.102	−0.093
September	−0.494	−0.835 to −0.153	0.005	−0.154	−0.545	−0.884 to −0.206	0.002	−0.193
October	−0.167	−0.545 to 0.212	0.389	−0.058	−0.166	−0.512 to 0.180	0.347	−0.053
November	−0.031	−0.372 to 0.311	0.860	−0.006	−0.074	−0.395 to 0.246	0.648	−0.023
December	−0.302	−0.687 to 0.083	0.125	−0.083	−0.317	−0.675 to 0.042	0.083	−0.096

*^a^No adjustment made for potential confounders*.

*^b^Adjusted for maternal and paternal age, annual household income, maternal history of depression and/or anxiety disorders, infantile gender, older brothers and sisters*.

## Discussion

We investigated whether season of birth affects emotional and behavioral regulation skills – higher effortful control and lower aggression – in 18-month-old infants using a Japanese birth cohort. The study demonstrated that effortful control was better for those born in spring (in particular April and May) and summer (in particular August) than in those born in winter. Furthermore, the propensity for aggression was lower in those born in spring (in particular April and May) than in those born in winter. These findings were observed even after considering maternal and paternal age, annual household income, maternal history of depression and/or anxiety disorders, infantile gender, and older siblings, thus suggesting that spring and summer births are associated with increased emotional and behavioral regulation skills during early infancy. The current findings corroborate previous studies indicating that winter births are associated with impulsivity-related traits including higher novelty seeking behavior (9, 11) and lower agreeableness (13) during adolescence. However, to the best of our knowledge, no studies have reported similar results among young children at 18 months of age. This research provides novel empirical evidence and may have implications for the literature regarding season of birth and development of emotional and behavioral regulation during early infancy. Consequently, we discuss the results in terms of fluctuations or variability rather than a cyclic nature for season of birth.

Although the coefficients were statistically significant, the magnitude of the effects of birth season on effortful control and aggression in 18-month-old infants seemed to be weak regardless of inclusion or exclusion of covariates because of generally low standardized partial regression coefficients that range from −1 to 1. These findings can be interpreted in three ways. One explanation is that season of birth has limited influences on development of emotional and behavioral regulation skills at 18 months after births. Therefore, because our results may be due to chance, caution is needed in interpreting the role of season of birth in emotional and behavioral regulation skills in early infants.

The second explanation is that the findings may stem from the effect of season of measurement, rather than season of birth. Births in spring and summer represent measurements in autumn and winter taking place at 18 months after birth, respectively. In addition, our research site is located in the Northern Hemisphere (34°42′N 137°43′′E), and warmer months with an average temperature above 15°C are usually recorded from May to October, as seen in Table 5 (25). This suggests that the patriating infants who were born in May experience 12 warmer months by 18 months of age, compared to those born in winter. A recent study reported that warmer weather accelerated neurodevelopment during early infancy in general (18). These facts suggest that infants have higher effortful control and lower aggression if measurements occur in autumn and winter than in other seasons. Therefore, it is possible that our results simply reflect season of measurement variations in emotional and behavioral regulation during infancy. However, to an extent, this possibility can be excluded because those born in August (a part of summer) and September (a part of autumn) displayed higher effortful control and lower aggression in 18-month-old infants.

**Table 5:** **Monthly change in temperature, humidity, and sunshine rate from 1985 to 2015 in Hamamatsu, Japan (25)**.

	Mean temperature (°C)	Mean maximum temperature (°C)	Mean minimum temperature (°C)	Mean humidity (%)	Mean sunshine rate (%)
January	6.0	15.5	–1.3	58.1	64.5
February	6.8	17.8	–1.0	57.5	60.1
March	9.9	20.5	0.7	59.8	53.6
April	14.7	25.0	4.5	64.6	51.5
May	18.8	28.1	10.5	71.2	46.1
June	22.1	31.6	15.3	78.3	34.5
July	25.9	35.0	19.5	80.6	41.2
August	27.2	35.5	21.1	77.7	53.9
September	24.4	33.4	16.4	75.6	45.5
October	19.1	28.2	10.6	70.5	48.5
November	13.7	23.2	4.7	65.9	55.4
December	8.5	18.4	0.6	61.0	65.8

The third explanation, which we consider most likely, is that season of birth may be associated with emotional and behavioral regulation during infancy via other factors that we did not investigate in this study. Such factors that connect season of birth with effortful control and aggression during infancy include maternal postpartum depressive symptoms and parenting style. It has been suggested that childbirth in winter is a risk factor for maternal postpartum depressive symptoms (26). Maternal postpartum depressive symptoms were also associated with less responsiveness to child distress, unsatisfactory breast-feeding patterns, and undesirable sleep practices in the infant (27). Thus, mothers with new babies who were born in winter may be more likely to experience postpartum depressive symptoms than other mothers, which consequently may result in such undesirable parenting. As a result, infants who have mothers displaying undesirable parenting may have lower levels of effortful control and higher levels of aggression. However, unfortunately, we have no data regarding this possibility. In order to test our interpretation, it is important to examine the effects of maternal postpartum depressive symptoms and parenting style.

Although we found that early infants who were born during warmer seasons had higher effortful control and lower aggression, the exact biological mechanisms are still unknown. If season of birth predicts higher effortful control and lower aggression, this may stem from seasonal variation of serotonin (5-HT) activity, as suggested by previous research (9). Previous reviews have suggested that an increase in 5-HT concentrations in cerebrospinal fluid is associated with less impulsive and aggressive behavior (28, 29). Seasonality of 5-hydroxyindoleacetic acid concentrations in cerebrospinal fluid, a major metabolite of 5-HT, also displayed a peak in spring and a trough in autumn (30, 31). Therefore, future studies need to investigate whether season of birth can influence 5-HT function, which may be related to emotional and behavioral regulation in infants at 18 months of age.

There are some limitations to this study that should be addressed. First, the generalizability of our findings based on the HBC Study may be restricted because the sample size was modest. Nevertheless, power analyses indicated that the number of participants in the four seasonal subgroups showed sufficient power (>80%) to detect significant effects (α = 0.05). Second, this study did not clarify the direction of causality between the variables because we did not evaluate changes over time in effortful control and aggression. Consequently, the findings that season of birth predicts effortful control and aggression in 18-month-old infants should be carefully interpreted. Effortful control and higher levels of aggression are expected to emerge from 1 to 3 years of age (3, 5); thus, it is interesting to repeatedly measure these variables in this age range in future studies. Third, we did not assess other traits regarding emotional and behavioral regulation skills including negative emotionality, novelty seeking behavior, and executive dysfunction, which may be also related to antisocial behavior in later developmental stages. Future research is needed to replicate the effects of birth season on other emotional and behavioral regulation measures in infants.

Despite these limitations, the current study has a number of methodological strengths, including (a) a general population sample in Japan, (b) a prospective study design from the second trimester of pregnancy to 18 months after birth, and (c) face-to-face interviews to measure effortful control and aggression. These strengths endorse the fact that our results are highly valid.

In conclusion, 18-month-old infants who were born in spring and summer had higher levels of emotional and behavioral regulation skills, e.g., greater effortful control and less aggression, compared with those born in winter. Further research should focus on the underlying mechanisms of the effects of birth season on development of emotional and behavioral regulation during infancy.

## Author Contributions

RA performed the statistical analysis and wrote the first draft of the manuscript. KT contributed to all aspects of this study, including the design, data collection and analysis, and drafting. NT provided administrative support and critical comments on the study design, data collection, and drafting. TH, YK, RN, CN, AO, YS, ST, and NM contributed to the preparation of the protocol, data collection, and interpretation of the results. All authors approved the final manuscript.

## Conflict of Interest Statement

The authors declare that the research was conducted in the absence of any commercial or financial relationships that could be construed as a potential conflict of interest.
